# Quantile Coarsening Analysis of High-Volume Wearable Activity Data in a Longitudinal Observational Study

**DOI:** 10.3390/s18093056

**Published:** 2018-09-12

**Authors:** Ying Kuen Cheung, Pei-Yun Sabrina Hsueh, Ipek Ensari, Joshua Z. Willey, Keith M. Diaz

**Affiliations:** 1 Department of Biostatistics, Mailman School of Public Health, Columbia University, New York, NY 10032, USA; 2 IBM Watson Research Center, Yorktown Heights, NY 10598, USA; phsueh@us.ibm.com; 3 Center for Behavioral Cardiovascular Health, Department of Medicine, Columbia University Medical Center, New York, NY 10032, USA; ie2145@cumc.columbia.edu (I.E.); kd2442@cumc.columbia.edu (K.M.D.); 4 Department of Neurology, Columbia University Medical Center, New York, NY 10032, USA; jzw2@cumc.columbia.edu

**Keywords:** citizen science, cluster analysis, physical activity, sedentary behavior, walking

## Abstract

Owing to advances in sensor technologies on wearable devices, it is feasible to measure physical activity of an individual continuously over a long period. These devices afford opportunities to understand individual behaviors, which may then provide a basis for tailored behavior interventions. The large volume of data however poses challenges in data management and analysis. We propose a novel quantile coarsening analysis (QCA) of daily physical activity data, with a goal to reduce the volume of data while preserving key information. We applied QCA to a longitudinal study of 79 healthy participants whose step counts were monitored for up to 1 year by a Fitbit device, performed cluster analysis of daily activity, and identified individual activity signature or pattern in terms of the clusters identified. Using 21,393 time series of daily physical activity, we identified eight clusters. Employment and partner status were each associated with 5 of the 8 clusters. Using less than 2% of the original data, QCA provides accurate approximation of the mean physical activity, forms meaningful activity patterns associated with individual characteristics, and is a versatile tool for dimension reduction of densely sampled data.

## 1. Introduction

Physical activity has been shown to improve cardiovascular health, reduce risk of mortality [1,2,3,4] and is an important component of primary prevention for many chronic diseases and conditions such as Type 2 diabetes and obesity [5,6]. Walking, in particular, is recognized as an easily accessible, convenient, and familiar mode of physical activity, and thus is an appealing strategy for the promotion of health and well-being. As such there is impetus for examining walking behaviors as a predictor of multiple health outcomes in ambulatory, community-dwelling adults. 

Advances in sensor technologies on wearable devices have enabled the continuous and accurate collection of step counts and other walking parameters over an extended period of time, thus providing a voluminous stream of data. The large amount of data provides an opportunity to better understand the daily physical activity patterns across populations. However, conventional analytical approaches focus on measuring physical activity patterns by predefined summary statistics such as total step counts and average minutes with activity on a given day. By summarizing physical activity at the daily level, however, these methods ignore between-day heterogeneity within a person, as they fail to capture the within-day patterns of activity. An understanding of within-day patterns of physical activity is of importance to facilitate individualized mobile experience, such as when push notifications and activity updates are being sent [7,8], and identifying changes in an individual’s daily routines, thereby facilitating tailored behavior intervention [9,10]. Given the broad use of step-counting trackers to monitor and improve physical activity [11,12,13,14,15,16,17,18,19,20,21,22,23,24,25], analyzing sensor data beyond predefined daily features thus can have significant public health impact.

Multivariate finite mixture modeling (MFMM) is a clustering method, whose purpose is to identify homogeneous subgroups wherein the number of subgroups is not assumed to be known in the analysis. The MFMM analysis is model-based, data-driven, and aims to produce subgroups with features arising from the same statistical distribution; dividing the data into an optimal number of subgroups based on specific criteria such as the Bayesian information criterion [26]. Clustering algorithms utilizing MFMM methods have been applied to identify dietary patterns [27,28] and physical activity patterns based on questionnaire data [29]. These algorithms often entail prespecifying only a small-to-moderate number of features as input variables, as the computational complexity grows exponentially with the addition of more features [30]. In the present context where the goal is to examine the within-day activity patterns, hundreds of physical activity inputs can be recorded from sensors throughout a day (e.g., minute-by-minute step counts), existing clustering algorithms may prove to be computationally infeasible without properly reducing the dimension of the data in a pre-processing step. 

Dimension reduction of sensor data continuously collected can be achieved by time series modeling of the data [31,32,33,34]. Typically, a time series is first transformed to a domain relevant to the scientific interest, and is then summarized by a few parameters (e.g., autocorrelation). These parameters in turn serve as input features in a clustering algorithm. In this article, we take a similar approach and propose a two-step method for analyzing sensor data as time series: the proposed method first transforms the daily physical activity data into a coarsened probability density function of quantiles of activity time, and then applies the MFMM analysis using the quantiles as input features. The method is thus called quantile coarsening analysis (QCA). This approach is motivated by the consideration that time of activity, as well as the amount of activity, is of primary interest in our application. As will be shown in *Statistical Analyses* below, the resolution or coarseness of dimension reduction can be set by users in accordance with the needs in their application; such flexibility distinguishes the proposed method from the traditional parametric modeling of time series data [35]. The purposes of this article are to demonstrate the feasibility of QCA in a data set of 21,393 time series of daily physical activity, and to examine its estimation properties under various degrees of coarseness.

## 2. Materials and Methods

### 2.1. Study Cohort

A single cohort, 12-month, intensive observational study was conducted in healthy adults with an objective to collect their personal daily stress and physical activity for associative analysis. The study was approved by Columbia University Medical Center’s (CUMC) institutional review board. All participants provided informed consent. Access to the study dataset and information about the study’s execution and materials is publicly available [36].

Potentially eligible participants were identified and screened at CUMC. The inclusion criteria were (i) aged 18 years or older; (ii) self-reported intermittent exerciser (i.e., exercise 6–11 times per month but did not have a regular workout schedule); (iii) having access to a personal computer and a smartphone. Exclusion criteria included individuals who (i) were unavailable for 12 continuous months; (ii) had serious medical comorbidity that would compromise their ability to engage in usual physical activity; (iii) had occupational work demands that required rigorous activity; or (iv) were unable to read and speak English. From January 2014 to July 2015, a total of 79 participants were enrolled and followed for 12 months. For the purpose of this article, we considered the physical activity data (described below). Details of enrollment, participant characteristics, and other association studies of stress level were previously reported [37]. Briefly, the data set for the present analysis consisted of 45 females and 34 males, with an overall mean age of 31.9 years (±9.5 years). In addition, we considered the following variables for association with physical activity: race/ethnicity (27 non-Hispanic whites vs. 52 others), education as an ordinal variable (13 having less than college vs. 34 completing college vs. 32 attaining graduate or professional degrees), employment status (64 full-time employed vs. 8 part-time), and partner status (32 having a partner or spouse vs. 45 being single).

### 2.2. Physical Activity 

Physical activity was monitored continuously for up to 12 months using a wrist-worn Fitbit activity monitor (Fitbit Flex) [38]. The Fitbit device, containing an accelerometer and an altimeter, tracks the wearer’s daily physical activity including steps, distance walked, and stairs climbed, and has been previously validated for measuring physical activity [39]. While the Fitbit devices (including the Fitbit Flex) have been demonstrated to have good validity for the objective measurement of physical activity, their accuracy has largely been reported for stepping-related physical activities (e.g., walking and running) [40]. Similar to other research-grade accelerometers, the Fitbit devices have poor accuracy for the measurement of cycling [41,42]. Furthermore, Fitbit instructs users to not swim with the Fitbit Flex because it is not waterproof [43], thus rendering it unable to assess swimming-based exercise.

Data from the devices were automatically uploaded to the Fitbit website whenever the device was within 15 feet of the base station, which was plugged into the participant’s own personal computer. Participants were instructed to sync and charge their device every 5–7 days to ensure no loss of activity data. The Fitbit accelerometer recorded data in one-minute epochs, starting at 12:00 a.m. and ending at 23:59 p.m. every day, yielding a time series of 1440 minute-epochs per day per individual. The raw minute-by-minute step count data were extracted from the manufacturer’s website using Fitabase (Small Steps Labs, San Diego, CA, USA) and were reduced using a novel QCA, described in *Statistical Analyses* below. Specifically, the raw data that was relevant to the present article included the step counts over one-minute intervals with a timestamp; data for each participant was converted to an “RDATA” file each associated with a unique participant ID. Based on the raw data, we calculated other predefined physical activity measurements, including total daily step counts, the duration in minutes of physical activity (PA, defined as having at least 50 steps in a minute), and activity midday (defined as the time when 50% of daily step counts were achieved).

### 2.3. Statistical Analyses

#### 2.3.1. Quantile Coarsening Analysis (QCA)

Let *Y*(*t*) denote the step counts at time t and *S*(*t*) = $\int_{0}^{t}Y\left(u\right)du$ be the cumulative activity up to time t $\in \left(0,\;t_{max}\right).$ Then

(1) $$T\left(p\right)\inf\left\{t:S\left(t\right)\geq p\;S\left(t_{max}\right)\right\}$$

denotes the time where 100*p* percent of the total activity has been achieved and will be referred to as the 100*pth* quantile of the activity time [44]. Specifically, activity midday is defined by the 50th quantile, *T*(0.5). The idea of QCA is to represent a time series *Y*(*t*) using multiple quantiles *T*(*p_j_*) for a prespecified set of *p*_1_ < *p*_2_ < … < *p_K_*, together with the total daily counts *S*(*t_max_*). The number *K* of quantiles determines the number of components used to represent *Y*(*t*), and hence controls the resolution or coarseness of the approximation. Define the *Kth* order quantile-coarse function of *Y*(*t*) as

(2) $$C_{K}Y(t)=\frac{S\left(t_{max}\right)}{\left(K+1\right)\left\{T\left(p_{j+1}\right)-T\left(p_{j}\right)\right\}}\;\mathrm{for}\;T\left(p_{j}\right)\leq t<T\left(p_{j+1}\right)$$

for *j* = 0, …, *K*, with the convention that *T*(0) = 0 and *T*(1) = *t_max_*. While *p_j_* can be any values between 0 and 1, we consider an evenly spaced grid, i.e., setting *p_j_* = *j*/(*K* + 1) for *j* = 1, …, *K*. It can be easily shown that the quantile-coarse function is invariant under the quantile transformation. That is to say, applying the quantile transformation to *C_K_Y*(*t*) will result in the same quantile representation as applying the transformation to the original *Y*(*t*), i.e., *C_K_*{*C_K_Y*(*t*)} = *C_K_Y*(*t*). As a result, there is no loss in information by converting between coarsened data and quantiles back and forth for any given K. 

Our data set consisted of a total of 21,393 days of minute-by-minute step counts from 79 study participants. For each daily time series, we evaluated the quantile-coarse function. The mean time series $\bar{Y}\left(t\right)$ of each cluster was then approximated by the corresponding mean quantile-coarse function $\bar{C_{K}Y}$(*t*). We calculated the integrated mean squared error:

(3) $$\int_{0}^{t_{max}}{\left\{\bar{C_{K}Y}\left(t\right)-\bar{Y}\left(t\right)\right\}}^{2}dt$$

to assess the accuracy of the quantile coarsening method under various coarseness values K.

#### 2.3.2. Cluster Analysis

We performed cluster analysis using MFMM with the quantile-coarse function *C_K_Y*(*t*) as input. Specifically, we considered *K* = 19 so that each time series Y(t) was represented by a total of 20 features, namely, *T*(0.05), *T*(0.10), *T*(0.15), …, *T*(0.95), and *S*(*t_max_*). Note that although we did not use common features such as PA minutes as direct inputs of the cluster analysis, these features were implicitly incorporated as they could be approximated from a quantile-coarse function. The number of clusters was determined based on the Bayesian information criterion [45]. After the MFMM analysis, physical activity features of each cluster were described using means and standard deviations, along with the mean time series $\bar{Y}\left(t\right)$ of each cluster.

#### 2.3.3. Association Studies

In order to identify important factors affecting a participant’s physical activity behaviors in terms of the identified clusters, association between the cluster membership and participant characteristics was assessed using generalized linear mixed model (GLMM) with a logit link in a univariate manner, with an adjustment for a weekend/weekday random effect nested within a subject random effect. For comparison purposes, we also examined the association of step-count based clusters with participant characteristics using the same GLMM approach.

## 3. Results

### 3.1. Physical Activity Clusters by Multivariate Finite Mixture Modeling

The MFMM analysis found an eight-cluster solution among the 21,393 series. Table 1 reports some summary physical activity measures in each cluster. The clusters were organized according to the average daily step counts, which were in concordance with PA duration. The least active cluster (Cluster 1) on average completed just below 1000 steps a day with 7.3 min in PA; this subgroup of activity either depicted a very sedentary pattern or effectively identified inactivity due to non-wear. The most active group (Cluster 8) had about 10,000 counts on average with 73 min in PA. The next two most active clusters (Clusters 6 and 7) had similar activity level to Cluster 8 and were within 1000 steps daily on average. However, activity midday in these clusters, ranging from noon to 3:00 p.m., occurred earlier than that of Cluster 8. While not as inactive as Cluster 1, Clusters 2 and 3 had low PA level when compared to the higher clusters, with different activity midday. Clusters 4 and 5 represented days of intermediate PA level. 

**Figure 1:**
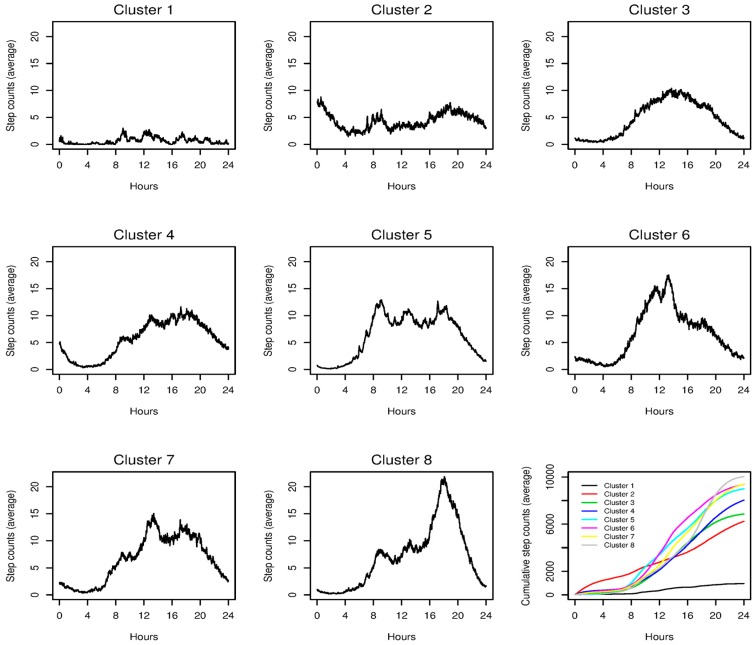
Mean activity of the 8 physical activity clusters by multivariate finite mixture modeling. Lower right: Superimposed cumulative step counts of the 8 clusters.

Figure 1 shows the mean activity curves of the clusters, and the superimposed cumulative activities of the clusters (lower right figure). These plots reveal additional cluster-defining features. Specifically, Cluster 2 was characterized by very early (i.e., late night) activity. Clusters 6 and 8 had peak activity averaged at around noon and 6:00 p.m. respectively, whereas Cluster 5 had multiple peaks throughout the day (at around 8:00 a.m., noon, and 5:00 p.m.).

### 3.2. Activity Patterns and the Weekends

Table 1 also shows the proportion of daily activity falling on a weekend for each cluster, and demonstrates a range across the eight groups with ≥40% of time series in Clusters 2 and 6 occurring on a weekend, and 16% in Cluster 5 being on a weekend. Generally, it is also noted that the time series in the inactive clusters (Clusters 1–3) tended to fall on weekends.

**Figure 2:**
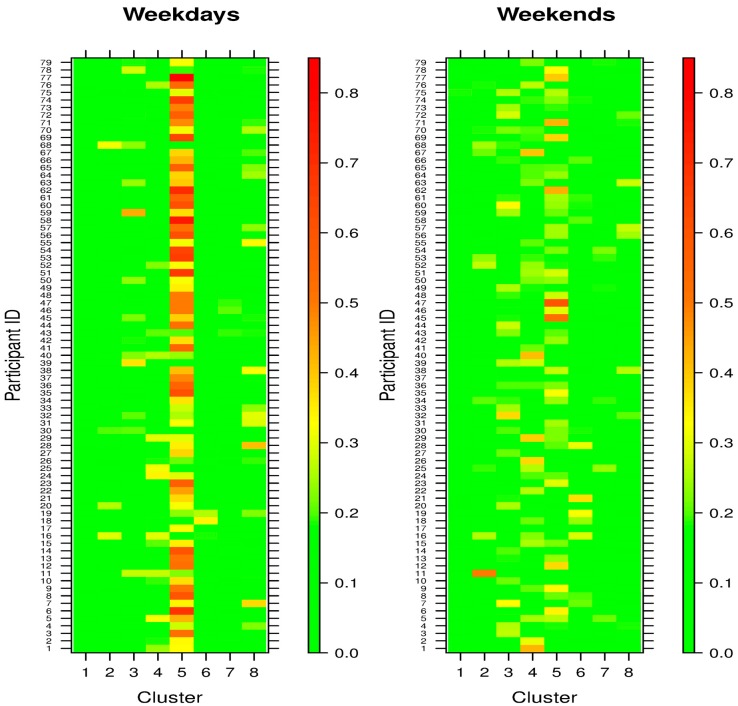
Heatmap of activity patterns of the 79 participants on weekdays and weekends. The color code indicates the proportion of days that a participant fell into each activity cluster.

Figure 2 further shows the PA patterns of the 79 participants were very different on weekdays and on weekends, with Cluster 5 being clearly a weekday phenomenon in most participants. It was consistent with the fact that Cluster 5 was characterized by spikes in activity around morning commute, lunch, and evening commute (Figure 1). At the same time, the heatmaps showed variations among the participants and that some did not follow this weekday/weekend differential (e.g., Participants 11 and 16). In addition, the PA patterns on the weekends were more dispersed than those on the weekdays, suggesting weekend activities were less structured and more heterogenous across participants.

### 3.3. Physical Activity Clusters and Participant Characteristics

Table 2 gives the association between each cluster and participant characteristics, in terms of odds ratio of falling into one activity cluster vs. the others using GLMM. In this cohort, employment and partner statuses were the most influential predictors of activity, each associated with 5 PA clusters. Specifically, Cluster 5 was highly significantly (*p* < 0.01) associated with being full-time employed and having a partner/spouse. Interestingly, the association between Cluster 5 and employment status was significant after adjusting for the weekend/weekday effect, suggesting that employment had a structural impact on an individual’s behaviors and habits beyond the physical constraint it has during a workweek. 

In contrast, Clusters 2 and 4, both having very early activity (Figure 1), were associated with singles with part-time jobs; having a younger age and receiving less education were also associated with these two clusters.

To a lesser extent, race/ethnicity was also predictive of an individual’s activity behaviors. Specifically, non-Hispanic whites were more likely to engage in physical activities consistent with Clusters 5 and 8, and less with Cluster 2. Finally, it is interesting to note that the inactive cluster (Cluster 1) was not associated with any particular characteristics.

### 3.4. Accuracy of Approximation

Table 3 gives the integrated mean squared errors of the quantile-coarse function using different values of K for estimating the mean activity of the 8 patterns. Accuracy improves as the original function $\bar{Y}\left(t\right)$ is represented with a larger number K of quantiles, with the initial improvement being most substantial. With K = 19, the mean squared error was about 3% on average of the error when daily activity was summarized using only the total daily counts (K = 0).

## 4. Discussion 

We have proposed a novel QCA for reducing dimension of data collected from wearable devices, and for representing data in conjunction with downstream analyses such as MFMM and association studies. The proposed method contributes to the analysis and management of wearable data in two ways. First, quantile transformation lends itself to making inferences about the time of activity, which could be useful in distinguishing individuals and days from a single individual with differing patterns of PA accrual. Using data from an intensive, 12-month observational study, we were able to identify 8 unique clusters (or subgroups) that characterized the various types of PA accrual patterns observed at the day-level and were able to link these clusters with participant characteristics that provided important contextual information regarding the observed patterns. For example, we observed a “worker” cluster (Cluster 5) associated with employment status wherein spikes in activity were observed around times of day that typically coincide with morning commute, lunch, and evening commute. We also observed active clusters that accrued much of the activity earlier or later in the day (Clusters 6–8), possibly reflective of morning or evening exercise. On the other hand, it is interesting to note that the most active pattern (Cluster 8) accumulates steps late in the day and is associated with full-time employment, suggesting these are intentional leisure-time physical activities. This is consistent with the literature that individuals who meet physical activity guidelines are those who engage in leisure-time physical activity [46]. In contrast, when we performed cluster analysis using total step counts only (i.e., not including time of activity as inputs), all but one cluster had an activity midday at 2:30 p.m. (Table 1). And as a result, we identified fewer and weaker association between the step-count based clusters and participant characteristics (Table 2); this analysis did provide nuances about the nature of activity, which in turn could be useful for developing applications of individualized intervention. 

Second, QCA facilitates large-scale data reduction, as quantile transformation requires only simple and scalable computations. We have demonstrated the method in a dataset of 21,393 time series (over 30 million minute-by-minute counts) from 79 participants for up to 1-year follow-up as a proof of concept. In real-life situations where deployment of mobile sensors such as Fitbit can occur at a much larger scale for a much longer duration, the large data volume will be a practical issue for storage and analyses and for the deployment of edge computing [47]. In a typical application, data are transmitted from the devices and stored externally on a server or in a cloud platform for specific analyses. Quantile coarsening in this context can be used as a data pre-processing step to minimize the volume of data transmission, storage, and persistence demand. As the size of the wearable devices tends to be small, their computational capacity is often limited. As such, continuous sensing may pose challenges to existing multi-modal analysis techniques using wearable devices. Since quantile transformation is easy to implement and can be computed independently of data from other individuals, simple scripts can be written to execute on the edge devices as well as on the server level. Depending on the purpose of the analysis, the end-user can specify the level of resolution in terms of the number K of quantiles needed. Our analyses show that the mean quantile-coarse function provides good approximation of the original mean function with only 20 data points per day per individual, representing less than 2% of the original amount of data (1440 data points). In addition, at the deployment time, QCA can also be applied on the incoming streams of data to compare to pre-stored cluster characteristics identified from the cluster analysis. This can lend support to the implementation of many other dynamic, just-in-time adaptive interventions that are key to persuasive reminder and sustainable behavioral changes [48]. 

The high volume of step count data offers the opportunities to tailor behavior intervention of each individual in a highly personalized manner. Specifically, we have created an activity behavior signature for each individual over time (Figure 2), which can serve as the basis of adaptive intervention. For instance, we could adapt the “dose” and time of push notifications if there are indications that an individual deviate from his/her own norm. The use of signature is broadly applicable to other behavioral intervention system such as centralized recommenders of health apps [49,50,51]. To allow for such tailored intervention, it is important to acknowledge individual behaviors are not monolithic, but heterogeneous. It is therefore important to note that our analysis goal was to identify clusters of daily activity as building blocks of each signature, as opposed to identify clusters of individuals. While within-day metrics (such as intensity and regularity [52]) have been examined to reflect enrich the heterogeneity in between-day activities of each individual, these approaches typically are semi-quantitative and are intended for visualization.

In the present article, we have shown the feasibility of QCA in a small cohort of relatively healthy individuals. The study design and analytical methods can be easily deployed to other populations. For example, the Northern Manhattan Study aims to assess risk factors for stroke and cardiovascular diseases, and has examined and analyzed the physical activity patterns of the cohort based on paper questionnaires [3,29]. It would be an interesting next step to follow up on these individuals to monitor and assess their mobility issues using wearables, and to provide additional information (signature) that contributes to cardiovascular risks.

We applied QCA to step count data. The method however is applicable to other data variety and supports monitoring of biometrics (e.g., heart rate, ambulatory blood pressure, etc.), location (e.g., indoor/outdoor), behaviors (e.g., medication adherence), exogenous factors such as weather, and user-input data via ecological momentary assessment. There is a growing trend towards self-monitoring on a daily basis with goals such as tracking health status, ameliorating exacerbations of chronic conditions, and avoiding episodic hospitalization; see [53,54,55,56,57] for example. As such, wearables devices are well-suited for this new approach to patient care, provided that they are capable of handling complex analysis efficiently (resulting in smaller and lighter devices with longer battery life). At the same time, we acknowledge that accelerometers are not capable of capturing some of the more common forms of aerobic exercise. Research- and commercial-grade accelerometers such as those made by Fitbit have poor accuracy for the measurement of cycling and cannot be worn while swimming due to not being waterproof [41,42]. However, given the versatility of the QCA, it shall provide useful unified analytical tools for the high data variety in multi-modal monitoring as sensing technologies advance.

## Figures and Tables

**Table 1:** Physical activity clusters by multivariate finite mixture modeling.

Cluster ID	1	2	3	4	5	6	7	8
N	409	1302	2285	2751	7819	1678	2326	2823
Daily step counts	961	6227	6855	8037	8999	9379	9396	10,038
Activity midday ^a^	11:30 a.m.	1:00 p.m.	2:00 p.m.	3:30 p.m.	2:00 p.m.	Noon	3:00 p.m.	5:00 p.m.
PA minutes ^b^	7.3	42.3	45.6	52.8	59.9	65.9	65.1	72.7
Weekend ^c^	37%	40%	39%	35%	16%	46%	30%	23%

^a^ Time of day when 50% of daily counts were achieved; time was rounded to nearest half-hour. ^b^ Duration (in minutes) with ≥50 counts per minute. ^c^ Percent of time series in the cluster being on a weekend.

**Table 2:** Association (odds ratio) of physical activity clusters and participant characteristics.

Cluster ID	1	2	3	4	5	6	7	8
Age ^a^	0.99	0.96 ***	0.99	0.98 *	1.02 *	1.01	1.00	1.01
Male (ref: Female)	0.77	0.94	0.80	1.37	0.86	1.04	1.02	0.95
NHW ^b^ (ref: others)	0.65	0.60 *	0.80	0.85	1.35 *	1.04	0.91	1.23 *
Education ^c^	1.02	0.66 **	1.01	0.75 *	1.15	1.17	0.91	1.11
Full-time (FT) (ref: Part-time, PT)	1.17	0.44 *	0.93	0.42 ***	3.49 ***	0.57 **	1.01	1.41 *
Being single (ref: Partner/spouse)	0.74	2.37 ***	0.76*	1.72 ***	0.65 **	1.02	1.19 *	0.85

^a^ Odds ratio per one-year increase in age. ^b^ NHW: Non-hispanic white. ^c^ Education as an ordinal variable: 0 = less than college; 1 = college graduate; 2 = above college. * ≤0.05, ** ≤0.01, *** ≤0.001.

**Table 3:** Integrated mean squared errors in estimating the mean activity of the eight clusters.

K	1	2	3	4	5	6	7	8
0 ^a^	563	3191	15,674	16,106	22,800	32,004	26,226	47,884
3	224	1690	5189	3926	13,473	8631	7356	15,360
9	59	609	880	908	2763	1519	2132	3084
19	29	255	253	342	676	506	626	826
39	19	131	98	135	181	188	211	237

^a^ = 0 corresponds to approximation using daily step counts only; activity is assumed to be uniform throughout the day.
